# Supplementary material for: Hot spot prediction in protein-protein interactions by an ensemble system
Source: BMC Syst Biol. 2018 Dec 31;12(Suppl 9):132. doi: 10.1186/s12918-018-0665-8 (PMC6311905; doi:10.1186/s12918-018-0665-8)
Supplement: Supplementary file 2 — All relevant MATLAB code. A simple Matlab implement of our predictor. (ZIP 19,742 kb) [file 12918_2018_665_MOESM2_ESM.zip › Additional file 2/Read ASA/ASA Property/BID/1UB4C.txt]

 NetSurfP - Protein Surface Accessibility and Secondary Structure Predictions

  

|  |  |  |
| --- | --- | --- |
|  |  | NetSurfP - Protein Surface Accessibility and Secondary Structure Predictions  Technical University of Denmark |

  


---

```
# For publication of results, please cite:
# A generic method for assignment of reliability scores applied to solvent accessibility predictions.
# Bent Petersen, Thomas Nordahl Petersen, Pernille Andersen, Morten Nielsen and Claus Lundegaard
# BMC Structural Biology 2009, 9:51 doi:10.1186/1472-6807-9-51
#
# Column 1: Class assignment - B for buried or E for Exposed - Threshold: 25% exposure, but not based on RSA
# Column 2: Amino acid
# Column 3: Sequence name
# Column 4: Amino acid number
# Column 5: Relative Surface Accessibility - RSA
# Column 6: Absolute Surface Accessibility
# Column 7: Z-fit score for RSA prediction
# Column 8: Probability for Alpha-Helix
# Column 9: Probability for Beta-strand
# Column 10: Probability for Coil
E G  1UB4C                  1    0.886  69.720  -1.462   0.003   0.003   0.994
E P  1UB4C                  2    0.607  86.190  -1.449   0.019   0.141   0.840
E H  1UB4C                  3    0.374  68.122  -0.238   0.021   0.451   0.528
E M  1UB4C                  4    0.298  59.610   0.273   0.022   0.552   0.426
B I  1UB4C                  5    0.116  21.552  -0.421   0.021   0.756   0.223
E H  1UB4C                  6    0.408  74.306   0.874   0.018   0.846   0.136
B S  1UB4C                  7    0.108  12.622   0.647   0.018   0.846   0.136
E S  1UB4C                  8    0.358  41.969   0.683   0.018   0.846   0.136
B V  1UB4C                  9    0.083  12.788  -0.330   0.018   0.846   0.136
E K  1UB4C                 10    0.353  72.571   0.792   0.018   0.846   0.136
E R  1UB4C                 11    0.456 104.538   0.592   0.021   0.756   0.223
B W  1UB4C                 12    0.172  41.366   0.562   0.021   0.451   0.528
E G  1UB4C                 13    0.373  29.363  -1.527   0.019   0.141   0.840
E N  1UB4C                 14    0.423  61.986  -1.320   0.018   0.047   0.935
B S  1UB4C                 15    0.327  38.324  -1.340   0.056   0.142   0.802
E P  1UB4C                 16    0.312  44.315   0.151   0.066   0.296   0.638
B A  1UB4C                 17    0.130  14.282  -1.171   0.074   0.484   0.442
B V  1UB4C                 18    0.074  11.374   0.140   0.022   0.552   0.426
E R  1UB4C                 19    0.297  68.036   0.335   0.069   0.386   0.545
B I  1UB4C                 20    0.071  13.117   0.841   0.118   0.150   0.732
B P  1UB4C                 21    0.070   9.919   0.630   0.354   0.048   0.598
E A  1UB4C                 22    0.304  33.545   0.806   0.938   0.007   0.055
E T  1UB4C                 23    0.416  57.727   1.022   0.970   0.001   0.030
B L  1UB4C                 24    0.039   7.232   0.361   0.970   0.001   0.030
B M  1UB4C                 25    0.057  11.346   0.256   0.970   0.001   0.030
E Q  1UB4C                 26    0.610 108.982   1.109   0.970   0.001   0.030
E A  1UB4C                 27    0.413  45.469   1.392   0.923   0.002   0.076
B L  1UB4C                 28    0.087  16.003   0.801   0.600   0.003   0.397
E N  1UB4C                 29    0.679  99.479   1.624   0.053   0.005   0.942
B L  1UB4C                 30    0.068  12.506   0.526   0.016   0.005   0.979
E N  1UB4C                 31    0.568  83.097   1.306   0.018   0.019   0.964
E I  1UB4C                 32    0.551 101.954   0.641   0.018   0.047   0.935
E D  1UB4C                 33    0.618  89.083  -0.486   0.005   0.015   0.979
E D  1UB4C                 34    0.288  41.501   0.886   0.004   0.085   0.910
E E  1UB4C                 35    0.506  88.416   1.671   0.002   0.816   0.182
B V  1UB4C                 36    0.079  12.204   1.330   0.001   0.900   0.099
E K  1UB4C                 37    0.369  75.800   1.425   0.001   0.959   0.040
B I  1UB4C                 38    0.059  10.897   1.270   0.001   0.959   0.040
E D  1UB4C                 39    0.425  61.185   1.574   0.001   0.959   0.040
B L  1UB4C                 40    0.117  21.514   1.035   0.000   0.983   0.017
E V  1UB4C                 41    0.499  76.681   1.052   0.001   0.900   0.099
E D  1UB4C                 42    0.759 109.314   0.287   0.005   0.015   0.979
E G  1UB4C                 43    0.437  34.400   0.027   0.005   0.015   0.979
E K  1UB4C                 44    0.317  65.269   1.482   0.002   0.816   0.182
B L  1UB4C                 45    0.054   9.924   0.886   0.001   0.959   0.040
B I  1UB4C                 46    0.113  20.942   1.175   0.001   0.959   0.040
B I  1UB4C                 47    0.042   7.807   0.055   0.001   0.959   0.040
E E  1UB4C                 48    0.313  54.716   1.942   0.001   0.900   0.099
B P  1UB4C                 49    0.145  20.632  -0.048   0.003   0.718   0.279
E V  1UB4C                 50    0.283  43.482   0.350   0.005   0.262   0.733
E R  1UB4C                 51    0.534 122.355   0.156   0.018   0.088   0.893
E K  1UB4C                 52    0.575 118.257   0.038   0.018   0.047   0.935
E E  1UB4C                 53    0.512  89.446  -0.230   0.018   0.019   0.964
E P  1UB4C                 54    0.423  60.052  -0.187   0.058   0.017   0.925
E V  1UB4C                 55    0.531  81.645   0.171   0.257   0.016   0.727
B F  1UB4C                 56    0.256  51.480   0.264   0.257   0.016   0.727
E T  1UB4C                 57    0.327  45.313   1.633   0.257   0.016   0.727
B L  1UB4C                 58    0.075  13.714   0.786   0.923   0.002   0.076
E A  1UB4C                 59    0.469  51.684   1.228   0.970   0.001   0.030
E E  1UB4C                 60    0.518  90.495   1.466   0.970   0.001   0.030
B L  1UB4C                 61    0.117  21.423   1.053   0.970   0.001   0.030
B V  1UB4C                 62    0.066  10.175   0.217   0.923   0.002   0.076
E N  1UB4C                 63    0.551  80.622   1.333   0.694   0.003   0.303
E D  1UB4C                 64    0.632  91.071   1.607   0.246   0.004   0.750
B I  1UB4C                 65    0.054   9.971   1.138   0.109   0.005   0.886
E T  1UB4C                 66    0.459  63.677   1.341   0.053   0.005   0.942
E P  1UB4C                 67    0.634  89.936   0.056   0.115   0.016   0.868
E E  1UB4C                 68    0.736 128.614  -0.327   0.109   0.005   0.886
E N  1UB4C                 69    0.511  74.810  -1.233   0.053   0.005   0.942
E L  1UB4C                 70    0.330  60.368  -0.096   0.058   0.017   0.925
E H  1UB4C                 71    0.415  75.434  -0.524   0.058   0.017   0.925
E E  1UB4C                 72    0.554  96.836  -0.783   0.115   0.016   0.868
E N  1UB4C                 73    0.513  75.103  -0.852   0.184   0.043   0.773
B I  1UB4C                 74    0.243  44.936   0.661   0.278   0.093   0.628
E D  1UB4C                 75    0.480  69.182  -0.888   0.191   0.086   0.723
B W  1UB4C                 76    0.272  65.320  -0.577   0.113   0.043   0.844
B G  1UB4C                 77    0.295  23.193  -1.449   0.115   0.016   0.868
E E  1UB4C                 78    0.595 103.877   0.935   0.339   0.016   0.645
E P  1UB4C                 79    0.485  68.864  -0.672   0.522   0.016   0.462
E K  1UB4C                 80    0.396  81.375   0.048   0.522   0.016   0.462
E D  1UB4C                 81    0.329  47.394  -1.124   0.522   0.016   0.462
E K  1UB4C                 82    0.594 122.186   0.549   0.430   0.016   0.555
E E  1UB4C                 83    0.513  89.639  -0.159   0.354   0.048   0.598
E V  1UB4C                 84    0.384  59.082   0.866   0.268   0.043   0.689
E W  1UB4C                 85    0.446 107.359  -1.203   0.003   0.003   0.994

Explain the output.  Go back.
```

---
